# Prevalence of suspected autism spectrum disorder and attention‐deficit hyperactivity disorder in a Japanese clinical sample with gambling disorder: A cross‐sectional study

**DOI:** 10.1002/pcn5.131

**Published:** 2023-08-13

**Authors:** Ryuhei So, Yoshitaka Sato, Nozomu Hashimoto, Toshi A. Furukawa

**Affiliations:** ^1^ Department of Health Promotion of Human Behavior Kyoto University Graduate School of Medicine/School of Public Health Kyoto Japan; ^2^ Okayama Psychiatric Medical Center Okayama Japan

**Keywords:** attention‐deficit hyperactivity disorder, autism spectrum disorder, comorbidity, gambling, gambling disorder

## Abstract

**Aim:**

Studies show gambling disorders are associated with attention‐deficit hyperactivity disorder (ADHD). The association between gambling disorders and autism spectrum disorder (ASD) has not been well studied, although ASD is often comorbid with ADHD and is associated with gaming disorder. The aim of this study was to estimate the prevalence of ASD and ADHD traits comorbid with gambling disorders and to examine the relationships between these traits and gambling problems in a clinical population.

**Methods:**

This single‐site cross‐sectional study was conducted at a Japanese addiction outpatient clinic treating gambling disorders. The Autism‐Spectrum Quotient (AQ) test and the Adult ADHD Self‐Report Scale (ASRS) were used to screen ASD and ADHD. The Problem Gambling Severity Index (PGSI) was used to assess the severity of the gambling problems. We calculated the prevalence of suspected ASD and ADHD with 95% confidence intervals (CI) based on a binomial distribution and performed univariate analyses to examine the relationships between the AQ and ASRS scores and the total PGSI score.

**Results:**

We included 97 of 197 potential participants. After screening the participants using the AQ and ASRS, we found that the prevalence of ASD traits was 29.8% (95% CI: 21.0%–40.2%), while the prevalence of ADHD traits was 26.0% (95% CI: 17.9%–36.2%). Univariate regression analyses revealed that the total AQ score was inversely associated with the total PGSI score. However, the total ASRS score and some ASRS subscores were positively associated with the total PGSI score.

**Conclusion:**

ASD and ADHD may be prevalent among patients with gambling disorders in clinical settings.

## INTRODUCTION

Gambling disorders are associated with many psychiatric comorbidities, including other addictive disorders, depression, anxiety, and attention‐deficit hyperactivity disorder (ADHD).^1^ Among the psychiatric comorbidities, ADHD has been extensively studied because gambling disorders were originally classified as an impulse control disorder.^2^ Previous studies show that 5%–20% of people with gambling disorders have ADHD,^3^ and comorbid ADHD is associated with increased severity of gambling problems.^4^

In contrast to ADHD, few studies have explored the relationship between gambling disorders and autism spectrum disorder (ASD), which is known to often co‐occur with ADHD. Initially, ASD traits, including low sensation seeking, were reported to protect against substance use disorders.^5^, ^6^ However, a recent systematic review qualitatively synthesized the results of 30 studies and demonstrated positive correlations between ASD traits and the severity of behavioral addiction, particularly gaming addiction.^7^ Only one study has reported the prevalence of ASD among treatment‐seeking individuals with gambling disorders.^8^ Similarly, only one study reported a positive correlation between the severity of ASD traits and gambling problems among nontreatment‐seeking young gamblers in general.^9^ In this study, the Pearson correlation coefficient between the total score on the short version of the Autism‐Spectrum Quotient (AQ),^10^, ^11^ a self‐administered screener for ASD known as the AQ‐10,^12^ and the severity of gambling symptoms was 0.239.

In recent years, the spread of loot boxes in gaming and online betting has obscured the boundaries between gaming and gambling. Under these circumstances, ASD, a consistent risk factor for gaming disorder in the extant literature, and ADHD^13^ should be explored relating to gambling disorders. Thus, we conducted a cross‐sectional study to estimate the prevalence of ASD and ADHD traits comorbid with gambling disorders and examined the relationship between these traits and gambling problems.

## METHODS

### Study design and settings

This cross‐sectional study was conducted at an outpatient clinic specializing in addictive disorders at the Okayama Psychiatric Medical Center in Japan. When we recruited participants, only the Okayama Psychiatric Medical Center provided specialized care for gambling disorders in the Okayama Prefecture. All patients visiting the outpatient clinic for the first time were invited to participate in the study.

### Participants

The participants were enrolled between June 2016 and July 2019. The inclusion criteria were (1) being aged at least 18 years old and (2) having a diagnosis of gambling disorder according to the diagnostic criteria of *The Diagnostic and Statistical Manual of Mental Disorders*, Fifth Edition (DSM‐5).^14^ For the exclusion criteria, potential participants deemed unable to fully answer the questionnaires by the researchers for reasons such as intellectual disability or dementia were excluded from the study.

### Data sources and measurements

The demographics and characteristics of the participants are listed in Table 1 and were collected from the pre‐examination records. The medical staff at the addiction outpatient clinic routinely conducted pre‐examination interviews according to the medical chart template. We did not use formal structured interviews or questionnaires for suicidal ideation and behavior. Instead, we used single questions during the pre‐examination interviews and recorded the patient's responses.

**Table 1:** Characteristics of participants.

Characteristic	*N* = 96^a^
Age (years)	38.50 (11.50)
Male	90/96 (94%)
Education (years)	14.09 (2.17)
Marital status	
Divorced	10/96 (10%)
Married	60/96 (62%)
Unmarried	26/96 (27%)
Employed	74/96 (77%)
Age at first gambling experience (years)	20.11 (4.66)
Age at onset of problem gambling (become aware of by oneself; years)	29.64 (9.53)
PGSI	15.90 (5.36)
Suicidal ideation	33/96 (34%)
Suicidal behavior	13/96 (14%)
HADS anxiety	8.90 (5.03)
HADS depression	8.76 (4.00)

Abbreviations: HADS, Hospital Anxiety and Depression Scale; PGSI, Problem Gambling Severity Index; SD, standard deviation.

^a^ Mean (SD); *n/N* (%).

In an outpatient clinic specializing in addictive disorders, psychiatrists diagnosed gambling disorders. Upon confirmation of the diagnosis, psychiatrists referred to the diagnostic criteria for gambling disorders in the DSM‐5. The Problem Gambling Severity Index (PGSI) was used to measure the severity of gambling problems as a continuous variable.^15^ The PGSI is a self‐administered questionnaire that includes nine items to assess gambling problems over the past 12 months. Each item's score ranged from 0 to 3. The total score is calculated by combining the scores of the nine items. Higher scores indicate more severe gambling problems. We have previously translated the PGSI into Japanese and examined its validity and reliability.^16^

The primary aim of this study was to estimate the prevalence of autistic traits and attention‐deficit hyperactivity traits associated with gambling disorders. We used the AQ^10^, ^11^ and Adult ADHD Self‐Report Scale (ASRS),^17^ respectively, to measure these traits.

The AQ is a self‐administered questionnaire comprising 50 questions and five subdomains, including social skills, attention switching, attention to detail, communication, and imagination. The total AQ score ranged from 0 to 50, with higher scores indicating stronger autistic traits. A total score of 33 was used to identify participants suspected of having ASD, according to a Japanese diagnostic accuracy study.^12^ We also used a cut‐off of 7 in the total score of the short form of the AQ‐10, which comprises 10 items from 50 items of the AQ, to retain comparability to some studies using the AQ‐10.^12^

The ASRS is a widely used self‐administered screening tool for ADHD, consisting of 18 items on a five‐point Likert scale and three subdomains: inattentiveness, motor impulsivity, and verbal impulsivity. Each item had a threshold for determining positive or negative results. The greater the number of items with responses above the threshold, the greater the functional impairment because of ADHD symptoms. In this study, we used a cut‐off score of 4 out of the first six items above the thresholds to determine participants with ADHD. We also used the total number of items with responses above the threshold to indicate levels of ADHD traits.

The severity of depression and anxiety was determined using the Hospital Anxiety and Depression Scale (HADS).^18^ The HADS is a 14‐item self‐administered scale of seven items each for depression and anxiety. The depression and anxiety score range from 0 to 21, with higher scores indicating greater depression and anxiety.

### Sample size

We assumed a 20% prevalence of ADHD comorbid with gambling disorder from a systematic review and meta‐analysis.^1^ We predetermined the target sample size as 100 to estimate the prevalence within a 95% confidence interval (CI) of ±10%. The expected prevalence of ASD comorbid with gambling disorder was not considered when calculating the sample size because of the lack of previous studies.

### Statistical analysis

Statistical analyses were conducted using R Version 4.2.1.^19^ We reported the demographic and clinical characteristics of the patients. The prevalence of ASD and ADHD was determined, and 95% CIs were calculated based on a binomial distribution. We performed univariate linear regression analyses using the total PGSI score as the dependent variable and each of the other variables, including the total scores and subscores of the AQ and ASRS, and screening results of the AQ and ASRS as the independent variables to examine the relationship between gambling severity and comorbid ASD or ADHD traits. Other univariate linear regression analyses were conducted by setting the total PGSI score as the dependent variable and using the same independent variables as in the above model.

## RESULTS

Informed consent and valid responses from 96 participants were obtained; 48.7% of patients with gambling disorders visited the outpatient clinic during the study period (*n* = 197). Table 1 presents the characteristics of the participants. Table 2 presents both the total scores and subscores of the AQ and ASRS.

**Table 2:** Suspected ASD and ADHD traits of participants.

Characteristic	*N* = 96^a^
AQ total	28.8 (6.58)
Social skill	4.86 (2.55)
Attention switching	5.60 (1.91)
Attention to detail	5.70 (2.01)
Communication	6.73 (2.24)
Imagination	5.91 (1.99)
ASRS total	4.67 (3.51)
Inattentive	3.01 (2.07)
Motor impulsivity	0.75 (1.21)
Verbal impulsivity	0.92 (1.04)

Abbreviations: ADHD, attention‐deficit hyperactivity disorder; AQ, Autism‐Spectrum Quotient; ASD, autism spectrum disorder; ASRS, Adult ADHD Self‐Report Scale.

^a^ Mean (SD).

Screening with the AQ and ASRS identified more than one‐quarter of the participants with ASD or ADHD traits (Table 3). When using all 50 items of the AQ, the prevalence of ASD traits was 29.8% (95% CI: 21.0%–40.2%). Using the short form of the AQ with 10 items (AQ‐10), as in some previous studies,^9^, ^20^ 36.2% (95% CI: 26.7%–46.8%) of the participants were determined to have ASD traits. Regarding ADHD traits, the prevalence among participants was 26.0% (95% CI: 17.9%–36.2%). In addition, 7.3% (95% CI: 3.2%–14.9%) had comorbid ASD and ADHD.

**Table 3:** Prevalence of suspected ASD and ADHD in participants.

Scale	Prevalence	95% CI
AQ	29.8% (28/94)	21.0, 40.2
ASRS	26.0% (25/95)	17.9, 36.2
AQ and ASRS	7.3% (7/96)	3.2, 14.9
AQ‐10	36.2% (34/94)	26.7, 46.8

Abbreviations: ADHD, attention‐deficit hyperactivity disorder; AQ, Autism‐Spectrum Quotient; ASD, autism spectrum disorder; ASRS, Adult ADHD Self‐Report Scale; CI, confidence interval.

Univariate regression analyses revealed significant associations between ASD and ADHD traits and the severity of problem gambling (Table 4). The total AQ score was inversely associated with the total PGSI score. In contrast, the total ASRS score and some ASRS subscores were positively associated with the total PGSI score. When dichotomizing the results of the AQ and ASRS using the cut‐offs, both AQ and ASRS positivity were significantly associated with a higher total score on the PGSI than AQ and ASRS negativity. Regarding other psychopathologies, including anxiety and depression, our analyses revealed inverse associations with AQ scores and positive associations with ADHD scores (Table 5).

**Table 4:** Univariate regression analyses between the total score of PGSI and scores of AQ and ASRS.

Characteristic	*β*	95% CI	*p*‐value
Raw scores			
AQ total score	−0.16	−0.33, 0.00	**0.049**
Social skill	−0.16	−0.59, 0.27	0.46
Attention switching	−0.49	−1.1, 0.08	0.09
Attention to detail	0.10	−0.44, 0.65	0.71
Communication	−0.65	−1.1, −0.18	**<0.01**
Imagination	−0.36	−0.91, 0.19	0.20
AQ‐10 total score	−0.26	−0.78, 0.27	0.34
ASRS total score	0.50	0.20, 0.79	**<0.01**
Inattentive	0.71	0.20, 1.2	**<0.01**
Motor impulsivity	1.5	0.64, 2.3	**<0.01**
Verbal impulsivity	0.82	−0.22, 1.9	0.12
Screening results by AQ and ASRS			
Both AQ and ASRS negative	Ref.		
Only AQ positive	−1.7	−4.3, 0.93	0.2
Both AQ and ASRS positive	5.8	1.8, 9.9	**<0.01**
Only ASRS positive	2.2	−0.80, 5.1	0.2

*Note*: *p*‐values less than 0.05 are in bold.

Abbreviations: ADHD, attention‐deficit hyperactivity disorder; AQ, Autism‐Spectrum Quotient; ASRS, Adult ADHD Self‐Report Scale; CI, confidence interval; PGSI, Problem Gambling Severity Index; Ref, Reference.

**Table 5:** Univariate regression analyses between scores of HADS and scores of AQ and ASRS.

	HADS anxiety			HADS depression		
Characteristic	*β*	95% CI	*p*‐value	*β*	95% CI	*p*‐value
AQ	−0.31	−0.45, −0.16	**<0.01**	−0.20	−0.32, −0.09	**<0.01**
Social skill	−0.35	−0.75, 0.05	0.08	−0.23	−0.55, 0.09	0.15
Attention switching	−1.1	−1.6, −0.62	**<0.01**	−0.60	−1.0, −0.19	**<0.01**
Attention to detail	−0.40	−0.91, 0.11	0.12	−0.18	−0.59, 0.22	0.38
Communication	−0.68	−1.1, −0.24	**<0.01**	−0.49	−0.84, −0.15	**<0.01**
Imagination	−0.47	−0.98, 0.04	0.07	−0.51	−0.91, −0.10	**0.01**
AQ‐10	−0.22	−0.71, 0.28	0.38	−0.22	−0.61, 0.18	0.29
ASRS	0.85	0.61, 1.1	**<0.01**	0.49	0.28, 0.70	**<0.01**
Inattentive	1.2	0.74, 1.6	**<0.01**	0.79	0.42, 1.1	**<0.01**
Motor impulsivity	2.7	2.1, 3.4	**<0.01**	1.5	0.85, 2.1	**<0.01**
Verbal impulsivity	1.3	0.36, 2.3	**<0.01**	0.48	−0.31, 1.3	0.23

*Note*: *p*‐values less than 0.05 are in bold.

Abbreviations: ADHD, attention‐deficit hyperactivity disorder; AQ, Autism‐Spectrum Quotient; ASRS, Adult ADHD Self‐Report Scale; CI, confidence interval; HADS, Hospital Anxiety and Depression Scale.

## DISCUSSION

This study estimated the prevalence of comorbid ASD, ADHD, or both traits in patients with gambling disorders in an addiction outpatient clinic. Additionally, we explored the associations between these traits and psychopathology. This study found that more than a quarter of the participants (29.8% with AQ and 36.2% with AQ10) were suspected of having ASD traits, which few studies have explored in clinical populations with gambling disorder. AQ scores were inversely associated with gambling problems, anxiety, and depression severity. The relationship between ADHD and gambling disorder has been extensively studied; 26.0% of the participants were suspected of having these traits. In contrast to ASD traits, higher ASRS scores were associated with more severe psychopathology. However, participants suspected of having both ASD and ADHD were associated with greater severity of gambling problems than those screening for either ASD or ADHD alone.

Interestingly, the high prevalence of suspected ASD with gambling disorders in the clinical sample exceeded the anticipated outcomes of this study. This discrepancy may reflect the high comorbidity of ASD and ADHD and the high comorbidity of ADHD and gambling disorder.^1^ Assuming that all potential participants were not suspected of having ASD, the prevalence of suspected ASD comorbid with gambling disorder in the clinical sample remained higher than the prevalence of suspected ASD in a previous Japanese study and the general Japanese population. In this least‐case scenario, the prevalence of comorbidities was 14.2% (28/197 with the AQ‐50) and 17.3% (34/197 with the AQ‐10), respectively. A cross‐sectional study in Japan^8^ reported that 2.5% of treatment‐seeking individuals with gambling disorders had ASD. This difference could arise from underestimation in a previous study that detected ASD by a retrospective medical chart review. In addition, a nationally representative survey in Japan reported that the prevalence of suspected ASD in the community was 5.1% (the AQ‐10).^20^

Previous studies have shown conflicting results regarding the association between suspected ASD and addictive disorders, including gambling. Although little is known about the relationship between suspected ASD and gambling, the inverse association in our results differed from that of a previous study, possibly because of differences in the population samples. A cross‐sectional study conducted in the United States^9^ included young nontreatment‐seeking gamblers, whereas this study involved a clinical population with gambling disorders across a broad age range. The associations observed among help‐seekers may be because of the intrinsic relationships among people with or without ASD. However, they can also be confounded by help‐seeking behaviors among people with or without ASD. Specifically, ASD traits may inherently protect against gambling problems, or individuals with ASD traits may be more likely to seek help from psychiatrists earlier than those without such traits.

Our results on ADHD and gambling disorders are consistent with those in the existing literature. The crude prevalence of suspected ADHD among participants was 26.0%. Under the least‐case scenario, as mentioned above, the minimum prevalence of comorbidities was 12.7% (25/197). The estimated prevalence range in this study was comparable to that of a previous study that used a self‐report questionnaire to diagnose ADHD^21^ and studies that used formal interviews.^3^, ^22^ The positive associations between ADHD severity and gambling problems are similar to those reported in previous studies.^22^, ^23^ In this study, motor impulsivity was the strongest factor associated with gambling. This finding also aligns with previous studies, including a large‐scale study^24^ exploring mediators between ADHD and problem gambling and a meta‐analysis synthesizing the effect sizes of behavioral and self‐reported motor impulsivity on pathological gambling.^25^

Despite the negative association between participants suspected of having ASD alone and gambling severity, participants suspected of having both ASD and ADHD showed a greater positive correlation with the severity of gambling problems than screening for either ASD or ADHD alone. These results imply an interaction between ASD and ADHD regarding the severity of gambling problems.

### Limitations

Some limitations of this study are related to the sampling process. The participants were recruited from a single addiction outpatient clinic. Moreover, we were unable to include 51.3% of the potential participants. These sampling method shortcomings undermine the validity and generalizability of the estimated prevalence of suspected ASD and ADHD in this study. Although the lower limit of prevalence based on the least‐case scenario provides a useful benchmark for comparison with findings from previous studies, further studies are needed to quantify the extent of these differences accurately.

However, the measurement issues may have other limitations. In this study, ASD and ADHD were not diagnosed using the DSM‐5,^14^ structured interviews using the Autism Diagnostic Observation Schedule (ADOS),^26^ or Conners' Adult ADHD Diagnostic Interview for DSM‐IV (CAADID).^27^ The self‐administered questionnaires used to assess ASD and ADHD traits in this study are usually less reliable and valid than formal structured interviews. Therefore, the prevalence reported in this study should be interpreted as the proportion of individuals suspected to have ASD, ADHD, or both. In addition, measurements with lower reliability can lead to greater variability in the results, which can reduce the precision of statistical estimates and statistical power. However, 95% CIs within ±10% for demonstrating the range of the prevalence in our study seem to be precise enough for practical purposes.

As for statistical power, though the totals score and some scores of AQ and ASRS were significantly associated with the severity of gambling problems, caution should be exercised that the lower reliability of AQ and ASRS might have inflated Type II errors. It must also be remembered that ASD and ADHD traits must have existed before the onset of the gambling disorder: Retrospective assessments of premorbid personality and other traits may be under recall bias.

## CONCLUSION

This study demonstrates that more than a quarter of the clinical population with gambling disorders may have ASD, ADHD, or both traits. Even when assuming that not all potential participants were screened for ASD, the prevalence of suspected ASD in this study was still higher than that in the general Japanese population. Furthermore, our results demonstrate that ASD traits are protective factors against severe gambling problems in a clinical setting while screening, as having both ASD and ADHD might increase the risk. For clinicians, the findings show that careful evaluation is recommended to screen for ASD and ADHD comorbid with gambling disorders and assess these traits' influence on gambling‐related problems. Since this study was a cross‐sectional study conducted at the commencement of therapy for gambling disorders, further studies are needed to examine the longitudinal relationships between gambling problems and ASD, ADHD, or both traits during therapeutic processes.

## AUTHOR CONTRIBUTIONS

Ryuhei So developed the study protocol, conducted the statistical analyses, and drafted the manuscript. Yoshitaka Sato and Nozomu Hashimoto collected the data and drafted the manuscript. Toshi A. Furukawa developed the protocol and supervised the whole process of the study. All authors revised the draft of the manuscript and approved the final version.

## CONFLICT OF INTEREST STATEMENT

Ryuhei So has received research grants from the Osake‐no‐Kagaku Foundation and speaker's honoraria from Otsuka Pharmaceutical Co., Ltd., Nippon Shinyaku Co., Ltd., and Takeda Pharmaceutical Co., Ltd. outside the submitted work. Ryuhei So also reports an employment position at CureApp Inc., which has developed software as a medical device. Toshi A. Furukawa reports personal fees from Kyoto University Original, grants and personal fees from Mitsubishi‐Tanabe, personal fees from SONY, grants and personal fees from Shionogi outside the submitted work. In addition, Toshi A. Furukawa has patents 2020‐548587 and 2022‐082495 pending, and intellectual properties for Kokoro‐app licensed to Mitsubishi‐Tanabe. Nozomu Hashimoto received speaker honoraria from Otsuka Pharmaceutical Co., Ltd. and Nippon Shinyaku Co., Ltd. Yoshitaka Sato declares no conflict of interest.

## ETHICS APPROVAL STATEMENT

The study protocol was approved by the institutional review boards of the Okayama Psychiatric Medical Center and Kyoto University.

## PATIENT CONSENT STATEMENT

We obtained written informed consent from all participants.

## CLINICAL TRIAL REGISTRATION

As this was not an interventional study, no prior registration was required.

## Data Availability

We did not obtain consent from the participants for sharing the data with external researchers; therefore, the data could not be provided.
